# Supplementary figures and images for: Minimal medical imaging can accurately reconstruct geometric bone models for musculoskeletal models
Source: PLoS One. 2019 Feb 11;14(2):e0205628. doi: 10.1371/journal.pone.0205628 (PMC6370181; doi:10.1371/journal.pone.0205628)

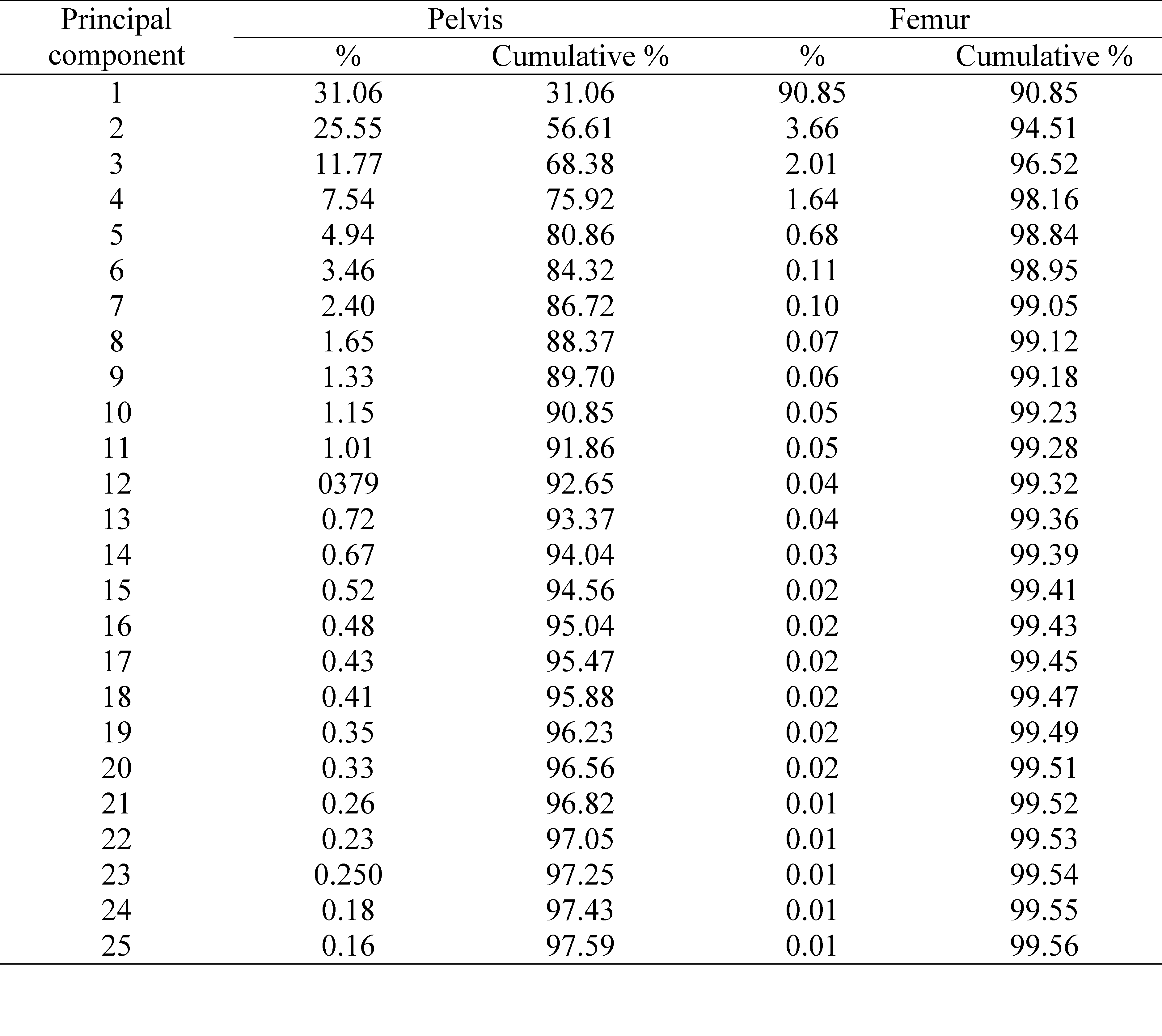

Supplement: S1 Table — (TIF) [file pone.0205628.s001.tif]

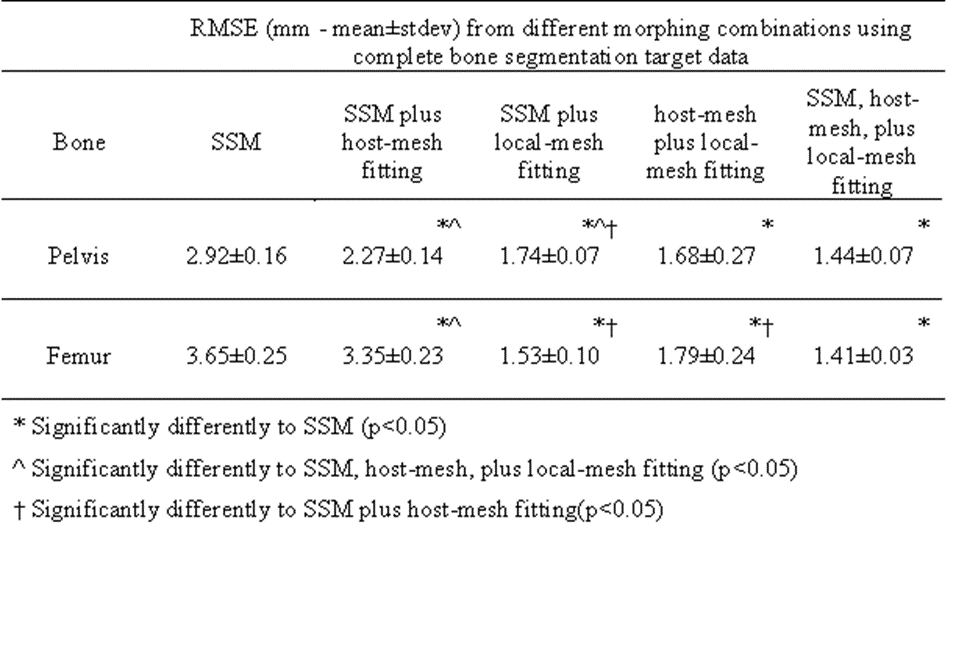

Supplement: S2 Table — Distance error (RMSE, mm) (mean ± standard deviation) of pelvis and femur reconstructions from complete bone segmentation for each participants of the subset (n = 5) through statistical shape modelling (SSM) and combinations of/with host-mesh and local-mesh fitting techniques. Differences (p<0.05) were investigated using repeated measures analysis of variance and selected pairwise comparisons with Bonferroni adjustment. (TIF) [file pone.0205628.s002.tif]

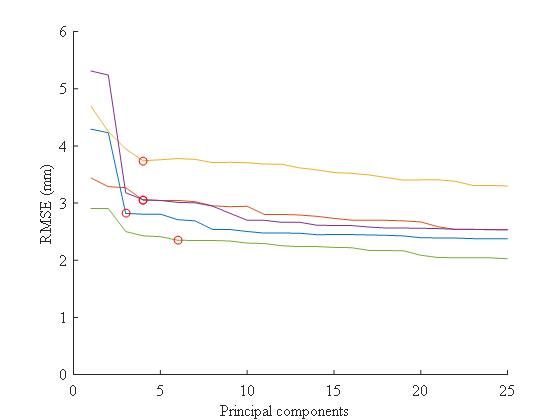

Supplement: S1 Fig — The average optimal number of principal components (red circles projected on the x-axis), for pelvis reconstruction of each participant included of the subset (n = 5) was 4.2±1.1. Optimal numbers were calculated by minimising the distance error (RMSE) and the least number of principal components. (TIF) [file pone.0205628.s003.tif]

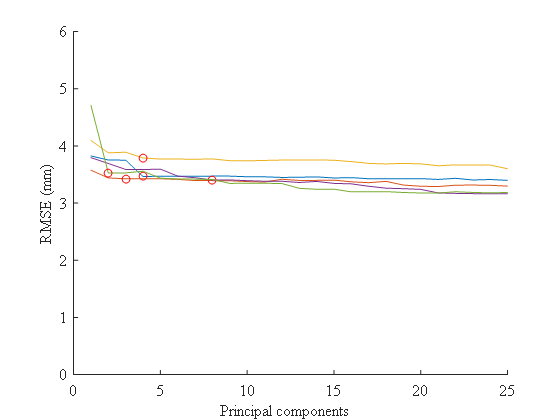

Supplement: S2 Fig — The average optimal number of principal components (red circles red circles projected on the x-axis), for femur reconstruction of each participant of the subset (n = 5) was 4.2±2.3. Optimal numbers were calculated by minimising the distance error (RMSE) and the least number of principal components. (TIF) [file pone.0205628.s004.tif]
