# Supplementary material for: Minimal medical imaging can accurately reconstruct geometric bone models for musculoskeletal models
Source: PLoS One. 2019 Feb 11;14(2):e0205628. doi: 10.1371/journal.pone.0205628 (PMC6370181; doi:10.1371/journal.pone.0205628)
Supplement: S1 File — Includes a list of the bone landmark marker, the method to calculate hip and knee joint centres, and the method to calculate scaling factors for each orthogonal direction of the pelvis and femur. (DOCX) [file pone.0205628.s005.docx]

**S5 Appendix**

Pelvis and femur motion capture-markers used for scaling

Right anterior superior iliac spine (RASIS)

Left anterior superior iliac spine (LASIS)

Right posterior superior iliac spine (RPSIS)

Left posterior superior iliac spine (LPSIS)

Lateral femoral epicondyle (LEC)

Medial femoral epicondyle (MEC)

Hip and knee joint centre estimation

Hip joint centre was estimated using a modified version of the Harrington regression equations which only uses pelvis width as an regressor (Harrington et al., 2007; Kainz et al., 2015; Sangeux, 2015).

Knee joint centre was defined as the midpoint between the medial and lateral epicondyles markers (Lamberto et al., 2017).

Pelvis scaling

Pelvis was scaled based on pelvis width and height; pelvis width was used for scaling in the medial-lateral and anterior-posterior directions, and pelvis height, defined as the perpendicular distance from a plane defined by the left and right ASIS markers and the midpoint between the left and right PSIS markers, to the HJC (mean distance between the values obtained from the left and right side), was used to scale the superior-inferior dimension of the pelvis.

Femur scaling

Hip joint centre to knee joint centre distance was used to scale the femur in all three dimensions.

References

Harrington, M. E., Zavatsky, A. B., Lawson, S. E. M., Yuan, Z., & Theologis, T. N. (2007). Prediction of the hip joint centre in adults, children, and patients with cerebral palsy based on magnetic resonance imaging. *Journal of Biomechanics*, *40*(3), 595–602. https://doi.org/10.1016/j.jbiomech.2006.02.003

Kainz, H., Carty, C. P., Modenese, L., Boyd, R. N., & Lloyd, D. G. (2015). Estimation of the hip joint centre in human motion analysis: A systematic review. *Clinical Biomechanics*, *30*(4), 319–329. https://doi.org/10.1016/j.clinbiomech.2015.02.005

Lamberto, G., Martelli, S., Cappozzo, A., & Mazzà, C. (2017). To what extent is joint and muscle mechanics predicted by musculoskeletal models sensitive to soft tissue artefacts? *Journal of Biomechanics*, *62*, 68–76. https://doi.org/10.1016/j.jbiomech.2016.07.042

Sangeux, M. (2015). On the implementation of predictive methods to locate the hip joint centres. *Gait & Posture*, *42*(3), 402–405. https://doi.org/10.1016/j.gaitpost.2015.07.004
